# *Culicoides* Latreille (Diptera, Ceratopogonidae) of Colombia: records from the collection of insects of medical importance from National Institute of Health

**DOI:** 10.3897/BDJ.12.e72511

**Published:** 2024-06-13

**Authors:** Erika Santamaria, Marco Fidel Súarez, Ricardo Ortiz Gallego, Patricia Fuya, Geraldine Páez, Catalina Marceló-Díaz

**Affiliations:** 1 Group of Entomology, National Institute of Health, Bogotá, Colombia Group of Entomology, National Institute of Health Bogotá Colombia; 2 Pan American Health Organization, La Paz, Bolivia Pan American Health Organization La Paz Bolivia; 3 Sistema de Información sobre Biodiversidad de Colombia, Bogota, Colombia Sistema de Información sobre Biodiversidad de Colombia Bogota Colombia; 4 Universidad Distrital, Bogotá, Colombia Universidad Distrital Bogotá Colombia; 5 Universidad INCCA de Colombia, Grupo de Investigación en Biotecnología y Medio Ambiente, Bogota, Colombia Universidad INCCA de Colombia, Grupo de Investigación en Biotecnología y Medio Ambiente Bogota Colombia

**Keywords:** Public Health, Ceratopogonidae, Oropouche virus infection, biting midges, entomology, species occurrence, Colombia, GBIF

## Abstract

**Background:**

The collection of insects of medical importance from the Instituto Nacional de Salud, INS (Bogotá, Colombia: https://www.ins.gov.co/Paginas/Inicio.aspx), was started in 1934 with the aim of being an institutional and national repository of the biodiversity of insects involved in vector-borne diseases of importance in public health. Today, the entomological collection includes more than 7,500 specimens.

The ceratopogonid insects are one group of Diptera that are represented in this collection. Within the Ceratopogonidae, the genus *Culicoides* Latreille, 1809 is relevant in public health because of the nuisance caused by their bites when they are presented in great abundance and because of their role as vectors of several agents (virus, protozoa and nematodes) that cause diseases to humans and to animals (Mellor et al. 2000, Mullen 2002). An overview of the Ceratopogonidae, represented in this collection, is presented here. A total of 801 individuals, mainly adults of the genus *Culicoides* (90%) are represented. The collection is the result of the effort of several researchers of the Group of Entomology at INS. These researchers collected ceratopogonids when they went to different transmission scenarios of vector-borne diseases in Colombia, with the purpose of making entomological characterisations including the processing, assembly and identification of the specimens in the laboratory.

**New information:**

New information about the geographical distribution of 39 species of the genus *Culicoides* in Colombia. All data have been uploaded to GBIF and are publicly available there.

## Introduction

The family Ceratopogonidae comprises a large group of small Diptera represented worldwide by at least 6,206 extant and 296 fossil species (Borkent and Dominiak 2020). It is composed of four subfamilies, with the subfamily Ceratopogoninae being the most diverse in both number of genera and species. The subfamilies Forcipomyiinae, Dasyheleinae and Leptoconopinae have a much smaller species diversity. Regarding the food preferences of adult insects in the family Ceratopogonidae, males are nectarific, while the nutrition of females is diverse (González et al. 2014). Females of some genera may be predatory, especially on small flying insects of similar or smaller size and may also be ectoparasites of other insects, sucking the haemolymph from Lepidoptera, Coleoptera, Odonata, phasmids, Neuroptera and Hemiptera, amongst others (Borkent 2004). However, the most studied groups are those that feed on blood from vertebrates, as are the genera *Culicoides* Latreille 1809, *Leptoconops* Skuse 1889 and *Forcipomyia* Meigen 1818, attacking mainly mammals, including humans and also birds, amphibians and reptiles (Szadziewski et al. 2011, González et al. 2014).

Focusing on the importance of the genus *Culicoides*, these insects are relevant in public health for the nuisance caused by their bites when there is a great abundance of them and for their role as vectors of various agents (viruses, protozoa and nematodes) that cause diseases to humans and animals (Harrup et al. 2015, Sick et al. 2019). In addition, depending on the degree of sensitivity of people to the saliva components of these biting midges, bites can cause dermatological reactions, ranging from papules and pustules as products of overinfection by scratching, to flaking with alteration of skin pigmentation (Forattini 1957, Sherlock 1965, Kettle 1995).

The most important disease transmitted by *Culicoides* to humans in the neotropics is the Oropouche virus (Negarnaviricota, Bunyaviridae), whose main vector is *Culicoidesparaensis* (Goeldi, 1905) (Walsh et al. 2021). In Colombia, sera of febrile patients with antibodies to the Oropuche virus have been found in the departamentos de Santander and Cundinamarca (Suárez et al. 2008). The species of *Culicoides* can also transmit to humans the nematodes of the genus *Mansonella* (Nematoda: Onchocercidae) (Undiano 1966, Mullen 2002). In Colombia, *Culicoidesinsinuatus* Ortiz & León, 1955 is suspected as one of the possible vectors of *Mansonellaozzardi* Manson 1897, in the Amazon Region (Tidwell and Tidwell 1982). In animals, the most notable disease transmitted by *Culicoides* is the bluetongue, caused by an Orbivirus (Duplornaviricota: Reoviridae) that affects cattle and sheep. In Colombia, the abundance of *C.insignis* Lutz, 1913 has been associated with the transmission of the bluetongue virus found in cattle in Antioquia (Homan et al. 1985).

In addition, the biting nuisance caused by *Culicoides* in some areas of Colombia had been characterised. In the rural area of nine municipalities of Boyacá, it was confirmed that the nuisance was caused by *Culicoidespachymerus* Lutz, 1914, with biting rates of up to 52 females/person in 5 minutes (Santamaria et al. 2008). Another species that has been recorded by biting humans is *Culicoidespuracensis* Wirth & Lee, 1967 in the Puracé moor, in Cauca (Rodriguez and Wirth 1986).

In relation to the knowledge of the biodiversity of species of the genus *Culicoides* in Colombia, three catalogues had been published reporting: the first, at least 88 species (Barreto 1986); the second, a list of 114 species confirmed and 66 species suspected may be in the country (Spinelli et al. 2009) and, finally, a third catalogue with 235 species of ceratopogonids, including 112 species of the genus *Culicoides* and 50 of the genus *Forcipomyia* Meigen, 1818 (Spinelli and Wolff 2016).

## General description

### Purpose

The resource presented here contains the description and quantification of ceratopogonids (Diptera, Ceratopogonidae) from the collection of insects of medical importance from the Group of Entomology at National Institute of Health (INS), Bogotá-Colombia. In total, 801 individuals make up this dataset, mainly adults of the genus *Culicoides* (90%). The species occurrence data are publicly available at GBIF (https://www.gbif.org/es/dataset/40a8aeb8-cf10-40b8-878d-54e6804ae9f9).

## Project description

### Title

Entomological research applied to the surveillance and control of vector-borne diseases (Investigación entomológica aplicada a la vigilancia y control de enfermedades transmitidas por vectores). This is a cross-sectional project from the Group of Entomology, in the framework of which, studies of outbreaks, epidemics and interdisciplinary studies of vector-borne disease transmission areas are continuously developed.

### Personnel

A significant contribution in the field collection, insect mounting process and identification of the ceratopogonids in this dataset was made by: between 1963 and 1970 (Suárez MF, Martínez E, Marinkelle CJ, Young DG, de Osorno F, Lee VH); and between 1971 and 1980 (Ferro C, Beltrán B, Guerra E, Strum H, Tidwell MA, de Rodríguez MC, Lozano R, Lee VH). More recently, between 2006 and 2010, the study "Ecological aspects and evaluation of methods of control of *Culicoidespachymerus* (Diptera, Ceratopogonidae)” was developed in the foothills of the Magdalena Valley of Boyacá, Colombia. In this study, ceratopogonids (adult and immature) were collected, some of which were included in the collection. Some researchers who participated in the study were Santamaría E, Ferro C, Carrasquilla MC, Zipa Y, Cabrera OL, Ahumada ML and Pardo R.

### Funding

National Institute of Health (Colombia) and Minciencias.

## Sampling methods

### Study extent

**Figure 1.**
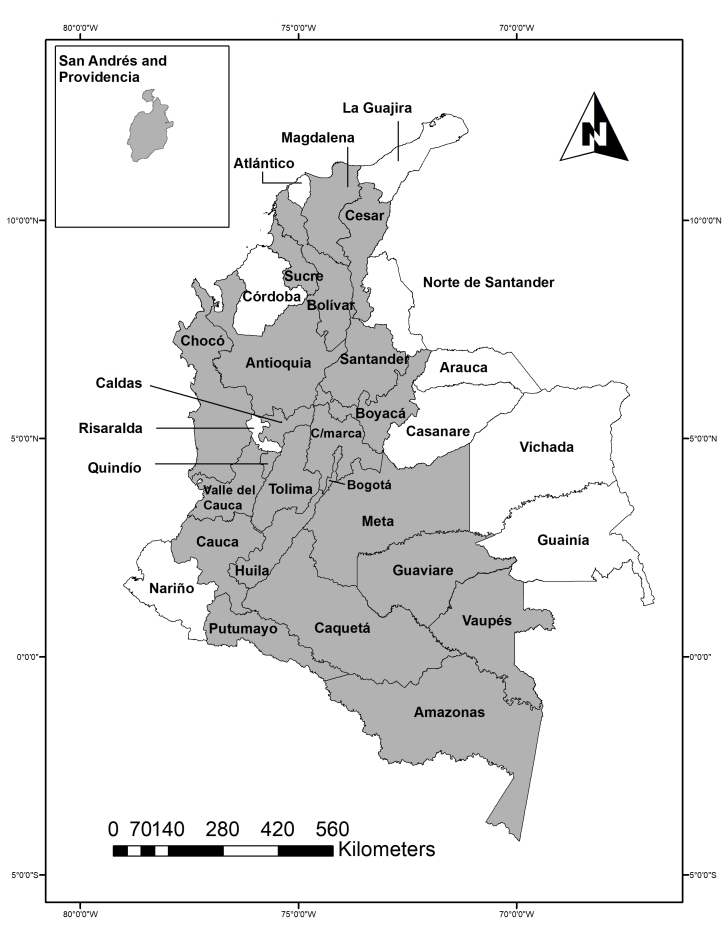
Colombian areas with Ceratopogonidae specimens present in the National Institute of Health (in grey).

Altogether 801 specimens are included in the dataset (Santamaria et al. 2020), mainly adults of the genera *Culicoides* (90%). It covers most of the Colombian territory, with records for 68.7% of the administrative areas in the country (Fig. 1). The regions with the highest number of records are Boyacá (217) and Tolima (166).

Boyacá, with an area of 23,189 km², is the administrative division mainly represented in the collection with more than 15 sampled locations. The greatest diversity of species are found in Boyacá (25 species), followed by Tolima (13) and Valle del Cauca (13). The administrative areas without records were Atlántico, Córdoba, La Guajira, Nariño, Norte de Santander, Risaralda, Arauca, Casanare, Guainía and Vichada.

### Sampling description

The ceratopogonid specimens in the collection of the National Institute of Health are the result of donations (e.g. paratypes of newly-described species) and collections made in field research carried out within the framework of vector-borne diseases study. Information about the sampling method of biting midges is available for 72.2% (n = 578) of collected specimens. The more common methods of sampling were human landing catches (43.4%) and light traps (28.7%). Other methodologies of sampling included Shannon trap, animal bait and CDC trap with CO_2_.

**Figure 3.**
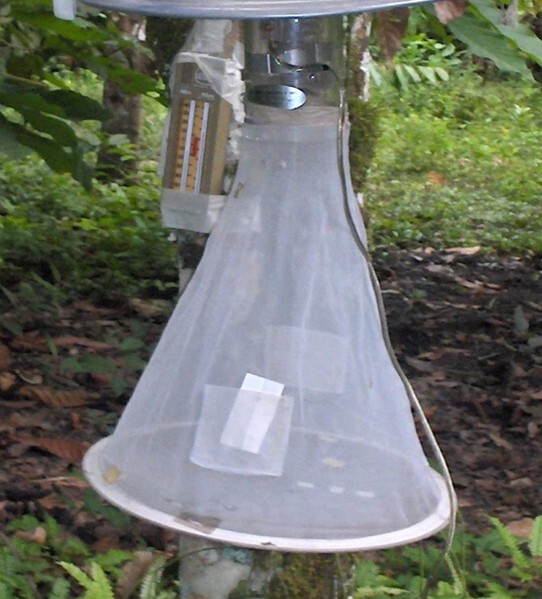
Light traps for the capture of *Culicoides* spp.

- Human landing catches: a volunteer exposed a hand and forearm, whereas another person collected, with the aid of a brush dampened in alcohol, the *Culicoides* as soon as the insects settled on the exposed skin (Santamaria et al. 2008, Fig. 2)

- Light traps: The vision of *Culicoides* and the ceratopogonids, in general, have not been well studied although its phototactic behaviour is of epidemiological importance (Allan et al. 1987). This behaviour is used widely to determine the presence, abundance and phenology (Kirkeby et al. 2013). Normally, the sampling is done by using CDC-type light traps with or without ultraviolet light installed near to stables or sheds where animals are located (Fig. 3).

Once the entomological sampling was carried out, the taxonomic identification of *Culicoides* was conducted, based on the wing pigmentation pattern and the original descriptions of the species (Wirth et al. 1988).

### Quality control

In all the *Culicoides* catches, previously-designed formats were used to record field collection information. The taxonomic identification was advised by external technical experts when necessary. The records of the dataset were confirmed and verified one by one.

Before the specimen is deposited in the collection, the curators of the collection from the Group of Entomology review the information associated with the specimen, i.e. the locality, geographical coordinates, sex, stage of development and taxonomy. The minimum of required information to include a specimen in the collection is related with the standard Darwin Core and is the same as the minimum information to be published in GBIF.

## Geographic coverage

### Description

**Figure 4.**
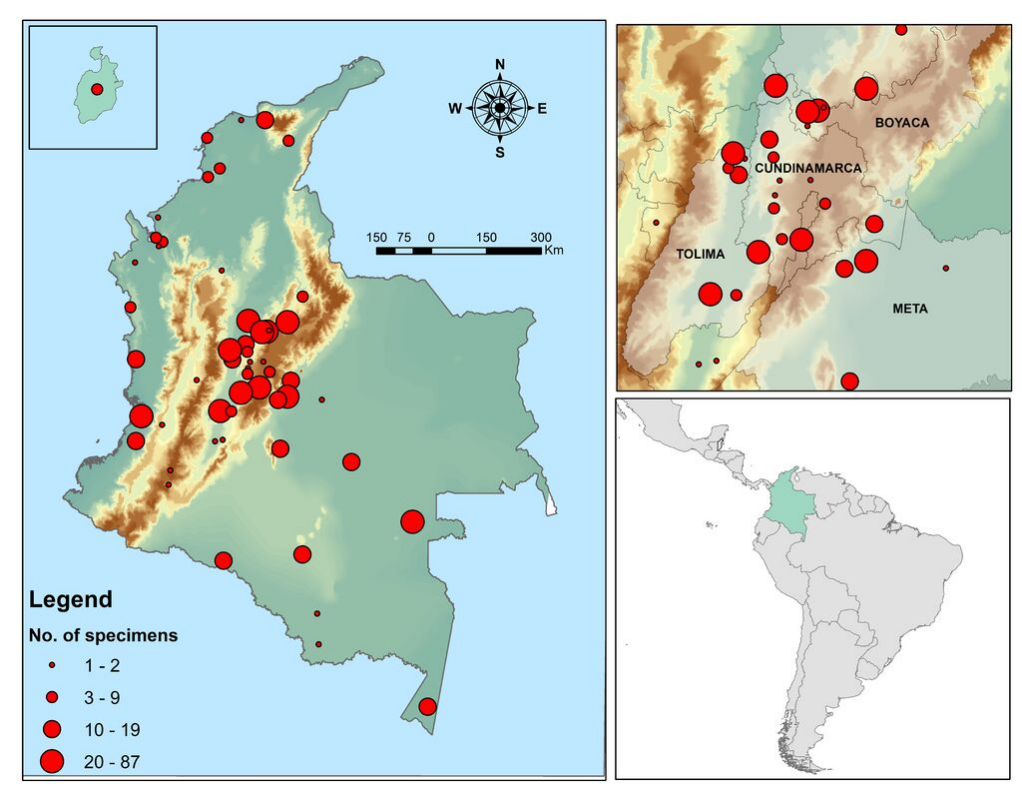
Geographic distribution and density per locality of specimens deposited in the collection at the National Institute of Health.

The collection of Ceratopogonidae includes 22 of the 32 administrative areas of Colombia. The specific collection location is available for 82.8% of the records. The digitised biting midge specimens are all from Colombia with the Andean natural Region at (63.9%), Amazonian Region (12.7%), Pacific Region (8.5%), Orinoquía Region (7.4%), Caribbean Region (4.5%) and Insular Region (0.6%), as shown in Fig. 4

From the data presented, there is only a record of altitude for adult specimens of *Culicoides* sp. collected in Monserrate, Bogotá (n = 19), the altitude recorded in the field being 3,230 m a.s.l. There is a register of altitude for the collections made in Boyacá and Cundinamarca .

The larvae (n = 55), collected in Boyacá (San Pablo de Borbur), were distributed in three groups: tribes Culicoidini (92.7%), Sphaeromiini (5.5%) and Ceratopogonini (1.8%), distributed in an altitudinal range between 437 and 439 m a.s.l.

The specimen occurrence data for all specimens are publicly accessible at GBIF: https://www.gbif.org/es/dataset/40a8aeb8-cf10-40b8-878d-54e6804ae9f9.

**New information on the distribution of *Culicoides* species for Colombia**

The following six *Culicoides* species were suspected in Colombia, but their specific location was unknown (Spinelli et al. 2009, Spinelli and Wolff 2016). Below are the new distribution reports in Colombia for each of these species:

*C.ignacioi* Forattini, 1957. Boyacá: Puerto Boyacá (n = 1); Cauca: López de Micay (n = 3); Putumayo: Puerto Leguízamo (n = 1) and Caquetá: Solano (n = 1).

*C.iriartei* Fox, 1952. Tolima: Coyaima (n = 2); Cundinamarca: Fusagasugá (n = 3) and Boyacá: San Pablo de Borbur (n = 1).

*C.jamaicensis* Edwards, 1922. Tolima: Coyaima (n = 3) and Cundinamarca: Medina (n = 1).

*C.neoparaensis* Tavares & Souza, 1978. Amazonas: Leticia (n = 6).

*C.rostratus* Wirth & Blanton, 1956. Meta: Villavicencio (n = 11) and Acacias (n = 2).

*C.volcanensis* Wirth & Blanton, 1959. Guaviare: San José del Guaviare (n = 1).

Additionally, this data paper updates the distribution in Colombia of another 33 species of ceratopogonids, most of them in the genus *Culicoides*. For each species, the new location report is presented as well as the number of specimens per locality.

*C.alahialinus* Barbosa, 1952. Antioquia: Apartadó (n = 2).

*C.balsapambensis* Ortíz & León, 1955. Boyacá: Pauna (n = 1).

*C.castillae* Fox, 1946. Tolima: Falan (n = 3).

*C.covagarciai* Ortiz, 1950. Boyacá: Togüí (n = 1); Meta: Acacias (n = 4) and Villavicencio (n = 4).

*C.dasyophrus* Macfie, 1940. Putumayo: Puerto Leguízamo (n = 2).

*C.debilipalpis* Lutz, 1913. San Andrés y Providencia: Providencia (n = 2); Meta: Villavicencio (n = 2); Cundinamarca: Sasaima (n = 1); Amazonas: El Encanto (n = 1), La Chorrera (n = 1); Boyacá: San Pablo de Borbur (n = 3) and Pauna (n = 2).

*C.diabolicus* Hoffman, 1925. Chocó: Bajo Baudó (n = 1); Meta: Villavicencio (n = 2).

*C.dicrourus* Wirth & Blanton, 1955. Tolima: Falan (n = 1).

*C.eublepharus* Macfie, 1948. Putumayo: Puerto Leguízamo (n = 1).

*C.filarifer* Hoffman, 1939. Boyacá: Puerto Boyacá (n = 2); Antioquia: Apartadó (n = 3); Putumayo: Puerto Leguízamo (n = 1).

*C.florenciae* Messersmith, 1972. Huila: Tello (n = 1), San Agustín (n = 1); Meta: Villavicencio (n = 1); Tolima: Mariquita (n = 1); Cauca: Lopez de Micay (n = 1).

*C.foxi* Ortiz, 1950. Boyacá: Puerto Boyacá (n = 2), Togüí (n = 19); Guaviare: San José del Guaviare (n = 7).

*C.furens* (Poey), 1853. Chocó: Bahía Solano (n = 3); San Andrés y Providencia: Providencia (n = 3); Magdalena: Sitionuevo (n = 2), Ciénaga (n = 3).

*C.gabaldoni* Ortiz, 1954. Antioquia: Turbo (n = 1).

*C.hylas* Macfie, 1940. Putumayo: Puerto Leguízamo (n = 1).

*C.insignis* Lutz, 1913. Bolívar: El Carmen de Bolívar (n = 3); Boyacá: Togüí (n = 2), San Pablo de Borbur (n = 2); Caquetá: Solano (n = 6); Cauca: López de Micay (n = 6); Chocó: Bajo Baudó (n = 9), Riosucio (n = 1); Cundinamarca: Medina (n = 16), Fusagasugá (n = 1); Tolima: Coyaima (n = 17).

*C.insinuatus* Ortíz & León, 1955. Tolima: Mariquita (n = 2), Armero (n = 1); Caquetá: Solano (n = 5).

*C.leoni* Barbosa, 1952. Boyacá: San Pablo de Borbur (n = 2); Bolívar: El Carmen de Bolívar (n = 1).

*C.leopoldoi* Ortiz, 1951. Antioquia: Apartadó (n = 1); Meta: Acacias (n = 1), Villavicencio (n = 1), Vista Hermosa (n = 1); Sucre: Tolú (n = 1); Chocó: Bajo Baudó (n = 1).

*C.mirsae* Ortiz, 1953. Antioquia: Carepa (n = 2), Necoclí (n = 2); Boyacá: Puerto Boyacá (n = 7), San Pablo de Borbur (n = 1), Pauna (n = 3); Cundinamarca: Caparrapí (n = 7), Útica (n = 9), La Mesa (n = 3); Tolima: Melgar (n = 19), Armero (n = 1).

*C.pachymerus* Lutz, 1914. Tolima: Armero (n = 11), Coyaima (n = 3), Mariquita (57), Prado (9); Antioquia: Turbo (n = 3); Boyacá: Muzo (n = 1), Pauna (n = 23), Puerto Boyacá (n = 2), San Pablo de Borbur (n = 7); Caldas: town near the river La Miel (n = 10); Cundinamarca: Caparrapí (n = 6); Vaupés: Mitú (n = 1).

*C.paraensis* (Goeldi), 1905. Boyacá: Pauna (n = 4), Puerto Boyacá (n = 24), San Pablo de Borbur (n = 24), Togüí (n = 1); Caldas: localidad cercana al rio La Miel (n = 2); Cesar: Valledupar (n = 5); Guaviare: San José del Guaviare (n = 3); Magdalena: Ciénaga (n = 11); Quindío: Armenia (n = 1); Santander: San Gil (n = 6); Tolima: Armero (n = 1).

*C.paucienfuscatus* Barbosa, 1947. Meta: Acacias (n = 1); Putumayo: Puerto Leguízamo (n = 1); Valle del Cauca: Buenaventura (n = 1); Vaupés: Mitú (n = 1).

*C.phlebotomus* (Williston), 1896. Sucre: Tolú (n = 3); Bolívar: Cartagena (n = 3); Huila: Neiva (n = 1).

*C.pifanoi* Ortiz, 1951. Boyacá: Puerto Boyacá (n = 1), Pauna (n = 3); Tolima: Mariquita (n = 1), Coyaima (n = 1); Cundinamarca: Caparrapí (n = 1).

*C.plaumanni* (Spinelli, 1993). Boyacá: Pauna (n = 1).

*C.pseudodiabolicus* Fox, 1946. Antioquia: Apartadó (n = 1); Cundinamarca: Caparrapí (n = 2), Sasaima (n = 1); Guaviare: San José del Guaviare (n = 1); Meta: Acacias (n = 2).

*C.pusillus* Lutz, 1913. Meta: Puerto López (n = 5), Acacias (n = 4); Caquetá: Solano (n = 1); Cesar: Valledupar (n = 4); Guaviare: San José del Guaviare (n = 1).

*C.trinidadensis* Hoffman, 1925. Huila: Neiva (n = 1); Tolima: Mariquita (n = 1), Melgar (n = 1); Boyacá: Puerto Boyacá (n = 1).

*C.venezuelensis* Ortiz & Mirsa, 1950. Cundinamarca: Bogotá (n = 7), La Calera (n = 4), Tabio (n = 2), Anolaima (n = 1); Boyacá: Puerto Boyacá (n = 1), Togüí (n = 2).

*Forcipomyiagenualis* (Loew), 1866. Cundinamarca: Medina (n = 2).

Forcipomyia (Lasiohelea) sp. Meta: Vistahermosa (n = 1); Guaviare: San José del Guaviare (n = 1).

*Dasyhelea* sp. Tolima: Coyaima (n = 2).

### Coordinates

-3.530059 and 13.35325 Latitude; -81.373939 and -70.045137 Longitude.

## Taxonomic coverage

### Description

**Figure 5.**
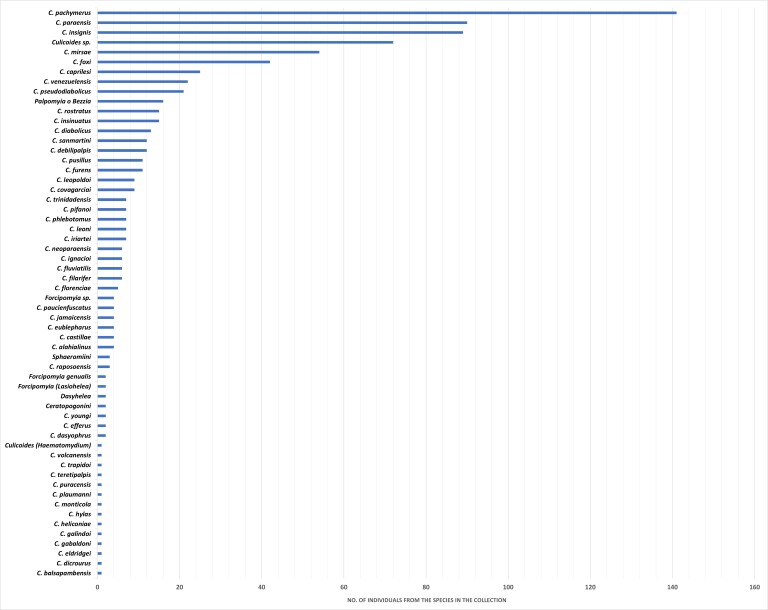
Species of Ceratopogonidae in the collection of the National Institute of Health.

From 801 specimens, 701 are identified at the species level, 77 at the genus level, 2 at the subgenus level and 21 at the tribe level (Fig. 5).

**Stage of development**: 90% of the records correspond to adults and 9.9% to larvae. The collection also includes the assembly of an egg.

**Paratypes**: As an important aspect to highlight, the dataset includes a total of 18 paratypes corresponding to the following species: *C.youngi* (n = 1), *C.teretipalpis* (n = 1), *C.trapidoi* (n = 1), *C.puracensis* (n = 1), *C.eldridgei* (n = 1), *C.raposoensis* (n = 3) and *C.sanmartini* (n = 10) (photos of some of these paratypes can be downloaded from the GBIF website). These paratypes were identified by Wirth W and Barreto P.

### Taxa included

**Table:** 

Rank	Scientific Name	Common Name
species	Culicoides (Oecacta) alahialinus Barbosa, 1952 https://www.gbif.org/species/1631166	Jején
species	*C.balsapambensis* Ortíz & León, 1955 https://www.gbif.org/species/1632551	Jején
species	*C.caprilesi* Fox, 1952 https://www.gbif.org/species/1632449	Jején
species	*C.castillae* Fox, 1946 https://www.gbif.org/species/1632388	Jején
species	C. (Anilomyia) covagarciai Ortiz, 1950 https://www.gbif.org/species/1632216	Jején
species	*C.dasyophrus* Macfie, 1940 https://www.gbif.org/species/1632305	Jején
species	C. (Haematomydium) debilipalpis Lutz, 1913 https://www.gbif.org/species/1631802	Jején
species	C. (Hoffmania) diabolicus Hoffman, 1925 https://www.gbif.org/species/1631578	Jején
species	C. (Mataemyia) dicrourus Wirth & Blanton, 1955 https://www.gbif.org/species/1632276	Jején
species	C. (Anilomyia) efferus Fox, 1952 https://www.gbif.org/species/1632141	Jején
species	C. (Haematomydium) eldridgei Wirth & Barreto, 1978 https://www.gbif.org/species/1631431	Jején
species	*C.eublepharus* Macfie, 1948 https://www.gbif.org/species/1632190	Jején
species	C. (Hoffmania) filarifer Hoffman, 1939 https://www.gbif.org/species/1631571	Jején
species	*C.florenciae* Messersmith, 1972 https://www.gbif.org/species/1632129	Jején
species	*C.fluviatilis* Lutz, 1914 https://www.gbif.org/species/168652777	Jején
species	C. (Hoffmania) foxi Ortiz, 1950 https://www.gbif.org/species/1631532	Jején
species	C. (Oecacta) furens (Poey), 1853 https://www.gbif.org/species/1632498	Jején
species	*C.gabaldoni* Ortiz, 1954 https://www.gbif.org/species/1632070	Jején
species	*C.galindoi* Wirth & Blanton, 1953 https://www.gbif.org/species/1632056	Jején
species	C. (Hoffmania) heliconiae Fox & Hoffman, 1944 https://www.gbif.org/species/1631484	Jején
species	C. (Hoffmania) hylas Macfie, 1940 https://www.gbif.org/species/1631469	Jején
species	C. (Hoffmania) ignacioi Forattini, 1957 https://www.gbif.org/species/1631451	Jején
species	C. (Hoffmania) insignis Lutz, 1913 https://www.gbif.org/species/1631477	Jején
species	C. (Haematomydium) insinuatus Ortíz & León, 1955 https://www.gbif.org/species/1631830	Jején
species	C. (Diphaomyia) iriartei Fox, 1952 https://www.gbif.org/species/1631880	Jején
species	C. (Drymodesmyia) jamaicensis Edwards, 1922 https://www.gbif.org/species/1631886	Jején
species	*C.leoni* Barbosa, 1952 https://www.gbif.org/species/1631772	Jején
species	*C.leopoldoi* Ortiz, 1951 https://www.gbif.org/species/1631757	Jején
species	C. (Diphaomyia) mirsae Ortiz, 1953 https://www.gbif.org/species/1631592	Jején
species	*C.monticola* Wirth & Lee, 1967 https://www.gbif.org/species/1631574	Jején
species	C. (Haematomydium) neoparaensis Tavares & Souza, 1978 https://www.gbif.org/species/1632322	Jején
species	*C.pachymerus* Lutz, 1914 https://www.gbif.org/species/1631521	Moscacilla
species	C. (Haematomydium) paraensis (Goeldi), 1905 https://www.gbif.org/species/1631418	Jején
species	*C.paucienfuscatus* Barbosa, 1947 https://www.gbif.org/species/1631442	Jején
species	C. (Macfiella) phlebotomus (Williston), 1896 https://www.gbif.org/species/1631220	Jején
species	*C.pifanoi* Ortiz, 1951 https://www.gbif.org/species/1631413	Jején
species	C. (Hoffmania) plaumanni (Spinelli, 1993) https://www.gbif.org/species/1631700	Jején
species	C. (Hoffmania) pseudodiabolicus Fox, 1946 https://www.gbif.org/species/1631808	Jején
species	C. (Avaritia) puracensis Wirth & Lee, 1967 https://www.gbif.org/species/1631965	Jején
species	C. (Avaritia) pusillus Lutz, 1913 https://www.gbif.org/species/163400920	Jején
species	C. (Cotocripus) raposoensis Wirth & Barreto, 1978 https://www.gbif.org/species/1631443	Jején
species	C. (Anilomyia) rostratus Wirth & Blanton, 1956 https://www.gbif.org/species/1631568	Jején
species	C. (Hoffmania) sanmartini Wirth & Barreto, 1978 https://www.gbif.org/species/1631523	Jején
species	*C.teretipalpis* Wirth & Barreto, 1978 https://www.gbif.org/species/1631460	Jején
species	C. (Anilomyia) trapidoi Wirth & Barreto, 1978 https://www.gbif.org/species/1631511	Jején
species	C. (Hoffmania) trinidadensis Hoffman, 1925 https://www.gbif.org/species/1631855	Jején
species	C. (Psychophaena) venezuelensis Ortiz & Mirsa, 1950 https://www.gbif.org/species/10814340	Jején
species	C. (Mataemyia) volcanensis Wirth & Blanton, 1959	Jején
species	C. (Haematomydium) youngi Wirth & Barreto, 1978 https://www.gbif.org/species/1631414	Jején
tribe	Ceratopogonini Kieffer, 1906	Jején
genus	*Dasyhelea* sp. Kieffer, 1911 https://www.gbif.org/species/1635616?occurrenceDatasetOffset=10	Jején
species	Forcipomyia (Forcipomyia) genualis (Loew), 1866 https://www.gbif.org/species/1633755	Jején
subgenus	*Forcipomyia* (Lasiohelea) Kieffer, 1921	Jején
tribe	Palpomyiini Enderlein, 1936	Jején
tribe	Sphaeromiini Newman, 1834	Jején
genus	*Culicoides* Latreille, 1809 https://www.gbif.org/species/1631084	Jején
genus	*Forcipomyia* Meigen, 1818 https://www.gbif.org/species/1633624	Jején

## Temporal coverage

**Data range:** 1905-5-30 – 2008-3-11.

### Notes

**Figure 6.**
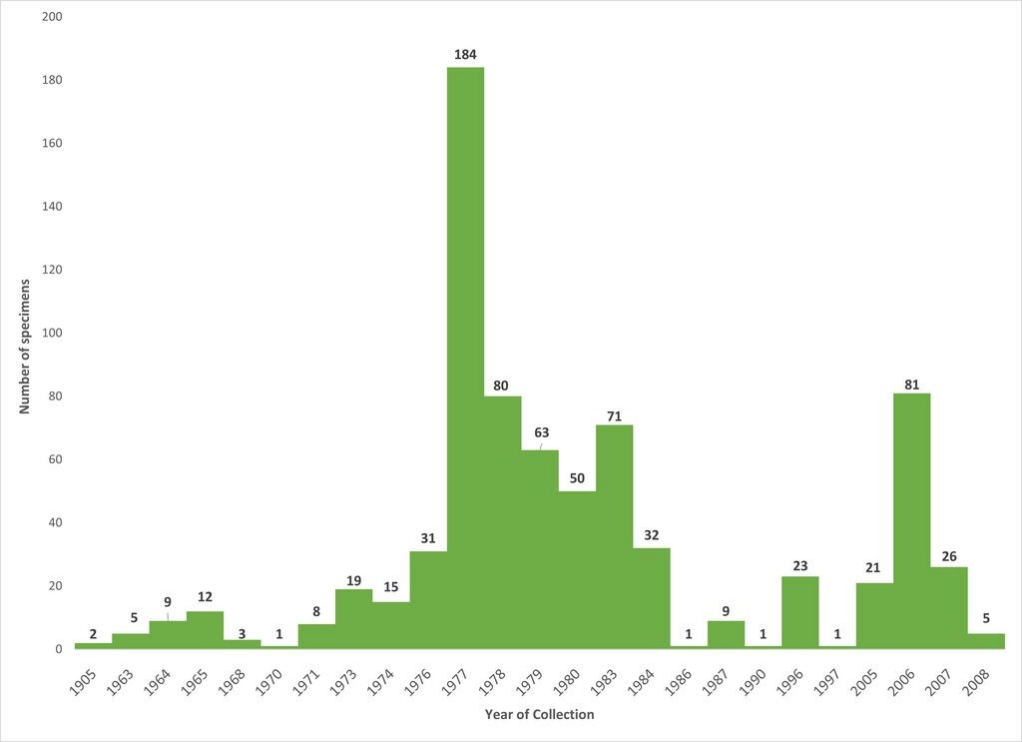
Temporal distribution of *Culicoides* specimens in the collection of the National Institute of Health (n = 753).

The *Culicoides* specimens, deposited in the collection of insects of medical importance from National Institute of Health, cover a timespan of 103 years between 1905 and 2008 (Fig. 6). This motivated us to designate this collection as an unique entity, which in fact is the result of heterogeneous entomological activities carried on by different collectors.

## Collection data

### Collection name

Collection of insects of medical importance

### Collection identifier

INS http://rnc.humboldt.org.co/admin/index.php/registros/detail/845

### Parent collection identifier

Unique National Registry of Biological Collections 056, Colombia

### Specimen preservation method

microscopic preparation

### Curatorial unit

Species collecting event

## Usage licence

### Usage licence

Other

### IP rights notes

Open Data Commons Attribution Licence (CC-BY-NC)

All data in the database can be freely used. Please cite this publication or the resource when using newly-presented data in your analyses.

## Data resources

### Data package title

Collection of insects of medical importance - Ceratopogonidae

### Resource link

https://doi.org/10.15472/5psmbm

### Alternative identifiers

https://www.gbif.org/es/dataset/40a8aeb8-cf10-40b8-878d-54e6804ae9f9

### Number of data sets

1

### Data set 1.

#### Data set name

Ceratopogonidae

#### Data format

Darwin Core

#### Character set

Occurrence

#### Description

Summary of ecological traits for 801 Biting Midges species.

**Data set 1.** 

Column label	Column description
occurrenceID	An identifier for the Occurrence (as opposed to a particular digital record of the occurrence). In the absence of a persistent global unique ID, one should construct one from a combination of identifiers in the registry so that the biological registry ID approximates a persistent identifier.
catalogNumber	An identifier (preferably unique) for the record within the dataset or collection.
basisOfRecord	The specific nature of the data record.
type	The nature or genre of the resource.
institutionCode	The name (or acronym) in use by the institution having custody of the object(s) or information referred to in the record.
institutionID	An identifier for the institution having custody of the object(s) or information referred to in the record.
collectionCode	The name, acronym, coden or initialism identifying the collection or dataset from which the record was derived.
collectionID	An identifier for the institution having custody of the object(s) or information referred to in the record.
language	Language of the resource.
scientificName	The full scientific name, with authorship and date information, if known.
scientificNameAuthorship	The authorship information for the scientificName.
taxonRank	The taxonomic rank of the most specific name in the scientificName.
higherClassification	A list (concatenated and separated) of taxa names terminating at the rank immediately superior to the taxon referenced in the taxon record.
kingdom	The full scientific name of the kingdom in which the taxon is classified.
phylum	The full scientific name of the phylum or division in which the taxon is classified.
class	The full scientific name of the class in which the taxon is classified.
order	The full scientific name of the order in which the taxon is classified.
family	The full scientific name of the family in which the taxon is classified.
verbatimTaxonRank	The taxonomic rank of the most specific name in the scientificName as it appears in the original record.
coordinateUncertaintyInMetres	The horizontal distance (in metres) from the given decimalLatitude and decimalLongitude describing the smallest circle containing the whole of the Location. Leave the value empty if the uncertainty is unknown, cannot be estimated or is not applicable (because there are no coordinates).
genus	The full scientific name of the genus in which the taxon is classified.
subgenus	The full scientific name of the subgenus in which the taxon is classified
georeferenceProtocol	A description or reference to the methods used to determine the spatial footprint, coordinates and uncertainties.
specificEpithet	The name of the first or species epithet of the scientificName.
lifeStage	The life stage (egg, larvae, adult) of the specimen of the Occurrence.
typeStatus	Status of the type. A list (concatenated and separated) of nomenclatural types (type status, typified scientific name, publication) applied to the subject. Controlled vocabulary of terms (HOLOTYPE, LECTOTYPE, ISOTYPE, SINTYPE, PARATYPE, NEOTYPE, EPITYPE, TYPUS).
sex	The sex of the specimen represented in the Occurrence.
identifiedBy	A list (concatenated and separated) of names of people who assigned the Taxon to the subject.
recordedBy	A list (concatenated and separated) of names of people, groups or organisations responsible for recording the original Occurrence.
georeferenceSources	A list (concatenated and separated) of maps, gazetteers or other resources used to georeference the Location, described specifically enough to allow anyone in the future to use the same resources.
year	The four-digit year in which the Event occurred, according to the Common Era Calendar.
month	The ordinal month in which the Event occurred.
day	The integer day of the month on which the Event occurred.
eventDate	The date-time or interval during which an Event occurred.
samplingProtocol	The name of, reference to, or description of the method or protocol used during an Event.
country	The name of the country or major administrative unit in which the Location occurs.
countryCode	The standard code for the country in which the Location occurs.
stateProvince	The name of the next smaller administrative region than country in which the Location occurs.
county	The full, unabbreviated name of the next smaller administrative region than stateProvince in which the Location occurs.
decimalLatitude	The geographic latitude (in decimal degrees, using the spatial reference system given in geodeticDatum) of the geographic centre of a Location.
decimalLongitude	The geographic longitude (in decimal degrees, using the spatial reference system given in geodeticDatum) of the geographic centre of a Location.
geodeticDatum	The ellipsoid, geodetic datum or spatial reference system (SRS) upon which the geographic coordinates given in decimalLatitude and decimalLongitude are based.
locality	The specific description of the place. Less specific geographic information can be provided in other geographic terms.
georeferenceRemarks	Notes or comments about the spatial description determination, explaining assumptions made in addition or opposition to the those formalised in the method referred to in georeferenceProtocol.

## Additional information

We consider it important to disclose the complete list of this historical collection of the Group of Entomology (INS) because of the importance in public health of Ceratopogonidae insects. We expect that this resource can serve as a reference for further studies of the biodiversity of these insects in Colombia and the Neotropics.

## Figures and Tables

**Figure 2.**
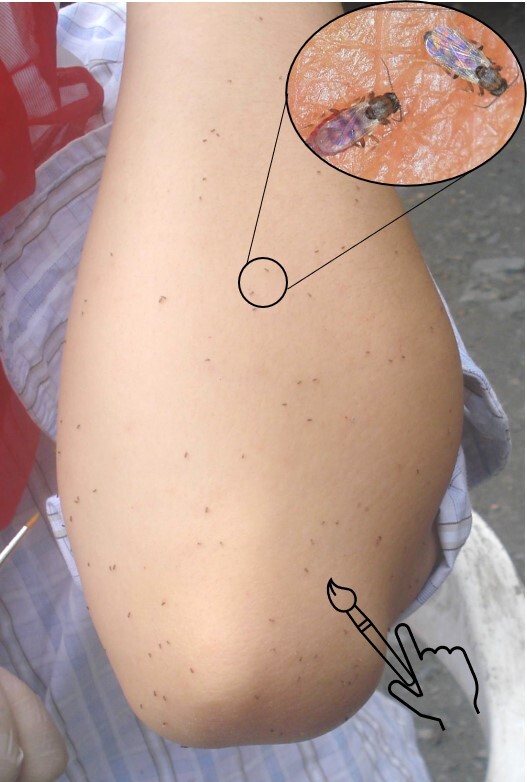
Human landing catches for the capture of *Culicoides* spp.
